# Supplementary material for: Genetic Approaches to Increase Arabinoxylan and β-Glucan Content in Wheat
Source: Plants (Basel). 2023 Sep 8;12(18):3216. doi: 10.3390/plants12183216 (PMC10534680; doi:10.3390/plants12183216)
Supplement: Supplementary file 1 [file plants-12-03216-s001.zip › plants-2564547-supplementary.pdf]

Table S1. β-glucan content of wheat species from selected publications from 1983 to 2020. Quantification was performed using the enzymatic method essentially as described by McCleary and Glennie-Holmes (1985) unless otherwise indicated.

Sample and source	Ploidy	β-glucan (%dw)	Details	Reference
**WHOLEMEAL**				
T. monococcum	Diploid	0.30		[159]
T. durum	Tetraploid	0.35		[159]
T. dicoccum	Tetraploid	0.35		[159]
T. dicoccum	Tetraploid	0.38	Mean of 5 varieties	[69]
Chinese elite wheat lines	Hexaploid	0.42	Mean value of range (27 lines)	[135]
Triticum spp	Tetraploid	0.42	Mean of 8 subspecies over 2 years	[114]
T. *aestivum*	Hexaploid	0.48	Mean of 3 varieties; calcoflour method	[171]
Triticum spp	Hexaploid & Tetraploid	0.48		[79]
Triticum spp	Tetraploid	0.48	Mean of all varieties over 2 years of sampling	[94]
Triticum spp	Diploid	0.48	Mean of all varieties over 2 years of sampling	[94]
Tetraploid wheat lines	Tetraploid	0.49	Mean value of range (88 lines)	[135]
Durum wheat	Tetraploid	0.49	cv. Svevo	[88]
T. durum	Tetraploid	0.53	Mean of 5 varieties	[69]
T. aestivum	Hexaploid	0.6		[172]
T. aestivum	Hexaploid	0.6		[89]
T. aestivum subsp. *Spelta*	Hexaploid	0.64	Mean of 5 varieties, 3 growing seasons, conventional and organic gorwth systems	[173]
T. spelta	Hexaploid	0.65		[159]
T. aestivum subsp. *Spelta*	Hexaploid	0.65	Mean of 90 genotypes over 3 years	[174]

Triticale	Hexaploid	0.66	Mean value of range	[135]
T. aestivum	Hexaploid	0.68	Mean of winter and spring varieties	[159]
T. aestivum	Hexaploid	0.7	Mean of values at two growth temperatures	[144]
T. aestivum	Hexaploid	0.7	Mean value of range (338 lines)	[135]
T. aestivum subsp. *aestivum*	Hexaploid	0.74	Mean of 5 varieties, 3 growing seasons, conventional and organic growth systems	[173]
T. aestivum	Hexaploid	0.75	Mean of 29 varieties	[69]
Wheat-barley addition	7Ds·7HL	0.75	Mean of values at two growth temperatures	[144]
CIMMYT synthetics	Hexaploid	0.76	Mean value of range (22 lines)	[135]
T. aestivum	Hexaploid	0.8	Mean of 10 lines	[175]
Triticum spp	Hexaploid	0.83	Mean of all varieties over 2 years of sampling	[94]
T. aestivum	Hexaploid	0.84	Mean over 3 years	[146]
Wheat-barley addition	7Bs·7HL	0.85	Mean of values at two growth temperatures	[144]
Wheat-barley addition	7As·7HL	0.95	Mean of values at two growth temperatures	[144]
Wheat-barley addition	7H	0.96	Mean of values at two growth temperatures	[144]
Wheat-barley addition		1.02	Mean value of range (6 lines)	[135]
Ae. crassa	Hexaploid	1.04	Mean of all varieties over 2 years of sampling	[94]
Primitive wheat	Diploid & Tetraploid	1.06	Mean value of range (35 lines)	[135]
Aegilops spp	Diploid	2.26	Mean of all varieties over 2 years of sampling	[94]
Ae. biuncialis	Tetraploid	3.67	Mean of 83 accessions over 2 years of sampling	[95]
Aegilops spp	Tetraploid	4.53	Mean of all varieties over 2 years of sampling	[94]
**ENDOSPERM**				
T. aestivum	Hexaploid	0.2	Endosperm flour	[176]
**ALEURONE**				
T. aestivum	Hexaploid	4.55	Mean of 2 different preparations	[130]

**BRAN**				
T. aestivum	Hexaploid	2.15	Red wheat	[93]
T. aestivum	Hexaploid	2.45	White wheat; mean of "bran 300" and "bran powder 50"	[93]
T. aestivum	Hexaploid	2.9		[130]
